# Revisiting noncoding RNAs: emerging coding functions and their impact on skeletal muscle development

**DOI:** 10.1038/s12276-025-01610-1

**Published:** 2026-01-08

**Authors:** Dandan Zhong, Jian Wang, Qi Li, Chuang Wang, Yuanyuan Huang, Yanhong Cao, Hui Li

**Affiliations:** 1 Guangxi Key Laboratory of Animal Breeding and Disease Control, College of Animal Science and Technology, Guangxi University, Nanning, China; 2 Guangxi Vocational University of Agriculture, Nanning, China

**Keywords:** Epigenetics, Non-coding RNAs, Bone development

## Abstract

Accumulating evidence has revealed noncoding RNAs (ncRNAs) as versatile regulators in skeletal muscle development, extending beyond their canonical roles as nontranslating transcripts. Recent advancements in proteomics and translatomics have demonstrated that ncRNAs containing cryptic open reading frames can encode peptides/proteins. Here we systematically evaluate computational tools and databases for predicting ncRNA-encoded products, dissect the molecular mechanisms underlying their translation and synthesize the current landscape of ncRNA-derived peptides/proteins identified in skeletal muscle across species. We further discuss their emerging roles in myogenesis and potential clinical implications for muscle-related disorders. By highlighting the dual functionality of ncRNAs as both regulatory RNAs and peptide/protein precursors, this work provides a comprehensive resource for understanding the expanding complexity of skeletal muscle development and proposes novel therapeutic targets for muscle diseases.

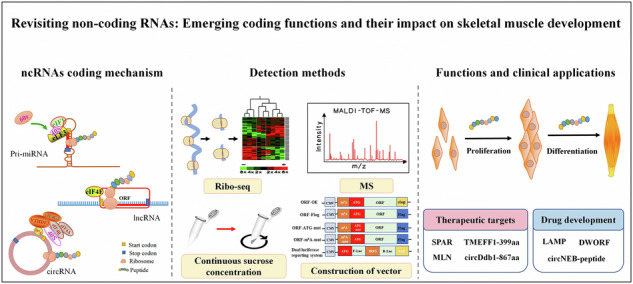

## Revealing peptide roles of noncoding RNAs in muscles

The study explores the role of noncoding RNAs (ncRNAs) in muscle development and disease. Here, we address the gap in understanding how these ncRNA-encoded peptides function in muscle biology. Researchers reviewed methods to predict and validate the protein-coding potential of ncRNAs. They used bioinformatics tools to identify small open reading frames within ncRNAs, which are sequences that can potentially code for proteins. Techniques such as ribosome profiling (Ribo-seq) and mass spectrometry were employed to confirm the presence of these peptides. The findings reveal that ncRNA-encoded peptides play crucial roles in muscle development and may offer new therapeutic targets for muscle diseases. For instance, some peptides regulate calcium transport in muscle cells, affecting contraction and growth. The study concludes that understanding ncRNA-encoded peptides could lead to novel treatments for muscle disorders.

This summary was initially drafted using artificial intelligence, then revised and fact-checked by the author.

## Introduction

The central principle of DNA > RNA > protein has been recognized by more and more researchers, and it has been shown that the dysregulation of this principle is closely related to the occurrence of diseases such as cancer. Proteins play a crucial role as the basic function and final product of genetic information, but the proportion of genomes that can code for them is about 2%^1^. Therefore, nonprotein-coding transcripts constitute the majority and are categorized into three types: microRNAs (miRNAs), long noncoding RNAs (lncRNAs) and circular RNAs (circRNAs), which are collectively referred to as noncoding RNAs (ncRNAs)^2^. miRNA is a type of small RNA approximately 22 nucleotides (nt) in length, mainly mediating post-transcriptional gene silencing by binding to mRNA targets^3^. lncRNA is usually longer than 200 nt and has a complex secondary structure, which can be involved in chromatin remodeling, transcriptional regulation and competing endogenous RNA networks^4^. circRNA is a closed circRNA formed by reverse splicing, lacking a 5′ cap and a 3′ poly(A) tail, and thus has higher stability^5^. Recent studies have revealed that ncRNAs are not ‘junk’ products but actively regulate myoblast proliferation and differentiation via transcription or competing endogenous RNA networks^6–8^. These ncRNAs are essential for skeletal muscle development, and their dysregulation contributes to muscle diseases such as muscular dystrophy.

The types of protein that are predicted to have coding functions are called annotated proteins and typically contain a typical open reading frame (ORF)^9^. However, ORFs are challenging to identify mainly due to their noncanonical protein-coding mode, arbitrary size restriction and initiation codon requirement^10^. Capping modification and polyadenylation occur at the 5′ and 3′ ends of primary miRNA (pri-miRNA) and lncRNA, respectively, which is similar to the way that RNA polymerase II participates in mRNA production^11^. Based on these features, it was hypothesized that the pri-miRNA and lncRNA might encode proteins as well. However, to initiate circRNA translation without end caps and polyadenylation tails, (*N*^6^-methyladenosine) m^6^A modifications or internal ribosome entry sites (IRES) play a key role^12^. Bioinformatic advances in recent years have uncovered several small ORFs (sORFs) at the genome-wide scale from RNAs classified as noncoding^9^. In deep RNA sequencing, sORFs are frequently observed throughout all transcript types^13^. As a result of ribo-seq, comprehensive, high-quality protein translation rates are measured at a genome-wide scale, as well as expression levels and abundances of proteins^14^. Further, to confirm the presence of a neglected peptide or protein, researchers have taken a proteomic approach (mass spectrometry, MS), which represents an unexpected library of biologically active proteins that are encoded by ncRNAs^15^. In recent years, important biological and pathological functions during skeletal muscle development have been studied by researchers, and a number of peptides or proteins encoded by ncRNAs have been identified. These findings challenge the traditional view of ncRNAs as nonfunctional transcripts.

This review focuses on ncRNAs with protein-coding potential, summarizing methods for predicting their coding ability and translational mechanisms. Specifically, we highlight functional peptides/proteins critical for muscle development and discuss their therapeutic potential in skeletal muscle diseases, aiming to provide theoretical insights and novel strategies for future research.

## Indentification of codifiable ncRNAs and valiation of coding peptides/proteins

### Bioinformatics

ORF is a DNA sequence extending from the start codon to the end codon, which has the potential to encode proteins and is a key unit in the transmission and expression of genetic information^16^. However, when predicting the ORF of transcripts, the threshold length of ORF that is less than 300 nt will filter the identification of some small ORFs. Therefore, a large number of transcripts are classified as ncRNA. However, some studies have shown that sORF does exist in lncRNAs and is translated into micropeptides (short peptides encoded by sORF, usually less than 100 amino acids, also known as ‘small peptides’) to perform their functions. For example, in the *Drosophila* heart there are two micropeptides translated through sORF to regulate calcium transport in the *Drosophila* heart and thus influence regular muscle contraction^17^. Therefore, exploring whether lncRNA and pri-miRNA have coding potential can predict whether they have ORF. For circRNAs, ORF with a cross junction in circRNA is considered to have coding potential. An interesting finding was that in addition to normal ORFs, infinite ORFs without stop codons were found in circEGFR. The authenticity of this infinite rolling loop translation was verified by ribosome enrichment and 3×FLAG tag fusion vectors^18^. Currently, ncRNA’s ORF and sORF can make predictions using algorithmic tools in bioinformatics. For example, the PhyloCSF tool mainly predicts ORF based on the conserved type of species sequence, whereas CNIT mainly predicts ORF based on ORF coverage and hexamer usage bias. In addition, proteomics and translationomics provide new evidence for unannotated ORFs and are currently being collected in multiple databases. Common algorithm tools and databases are presented in Table 1.

**Table 1:** Identification of codable ncRNA databases and tools.

Name	Website	Species	Content
ORF finder	https://www.ncbi.nlm.nih.gov/orffinder/	Universal	To evaluate the encoding capacity of RNA transcripts
PhyloCSF	https://github.com/mlin/PhyloCSF/wiki	Multispecies (any with genomic data)	PhyloCSF can be used for ORF and exon alignment
CNIT (Coding-Non-Coding Identifying Tool)^89^	http://cnit.noncode.org/CNIT/	Vertebrates (human, mouse, rat)	To evaluate the encoding capacity of RNA transcripts
sORFs.org^90^	http://www.sorfs.org	Primarily vertebrates (human, mouse)	A new database of sORFs was identified using ribosome sequencing analysis
SmProt^91^	https://smprot.biolead.ac.cn/	Multispecies	Micropeptide databases collected from literature mining, known databases, ribosome binding analyses, and MS
sORFfinder^92^	http://evolver.psc.riken.jp/	Original study in yeast	A package used to identify small ORFs with high coding potential
smORFunction^93^	https://www.cuilab.cn/smorfunction	Universal	A tool for predicting the function of small ORFs and microproteins
ARA-PEPs^94^	http://www.biw.kuleuven.be/CSB/ARA-PEPs	Plant (*Arabidopsis* *thaliana*)	A putative peptide library encoded by sORF in the Arabidopsis genome
PsORF^95^	http://psorf.whu.edu.cn/	Plants (35 species)	A unique comprehensive database of sORFs for 35 different plants
MetamORF^96^	https://metamorf.hb.univ-amu.fr/	Vertebrates (human, mouse)	Provide a repository of unique sORFs identified in human and mouse genomes by experimental and computational methods
OpenProt^97^	http://www.openprot.org	Eukaryotes	Contains all possible ORFs of more than ten codons from 30 species, accumulating supporting evidence for protein preservation, translation and expression
MiPepid^98^	https://github.com/MindAI/MiPepid	Universal	The DNA sequence of sORF was obtained and its micropeptide coding ability was predicted

### Ribo-seq

As a ‘factory’ for protein translation, ribosomes can convert nucleotide sequences on transcripts into specific amino acid sequences to form specific protein products. In 2009, Ingolia and colleagues introduced ribosome analysis^19^, known as ribosomal footprints, primarily by trapping ribosomes in RNA through translation inhibitors. The RNA that is not bound to ribosomes is digested using RNase and the remaining RNA is sequenced to obtain the RNA fragment being translated. This is more convincing for further proving whether lncRNA and pri-miRNA can be encoded. In fact, researchers identified 12 coding potential lncRNAs in colorectal cancer cells through Ribo-seq^20^. For circRNAs, the ribosome-bound fragment must be at the reverse splice, which is very stringent for codable circRNA screening. For example, 40 ribosome-related circRNAs have been identified through Ribo-seq and in human heart tissue^21^. Moreover, researchers have established a translatomics oriented circRNA database, the riboCIRC database, which mainly contains 3,168 Ribo-seq public data sets for six species. In recent years, the development of single-cell ribosomal profiling technology has enabled the study of the translatome at single-cell resolution, offering a novel perspective to uncover cellular heterogeneity and translational regulatory mechanisms^22^. By integrating microfluidics or single-cell sorting with ribosomal footprint sequencing, this technology allows the analysis of translational heterogeneity within cell populations and is expected to reveal differences in ncRNAs’ translational activity across cell types such as myosatellite cells and myoblasts.

### MS

Ribo-seq captures RNA fragments bound to ribosomes, thereby indirectly reflecting translation kinetics, but it cannot directly verify the existence of peptides/proteins. MS is a core technology for detecting proteins and micropeptides in the body. By detecting peptide molecules, it can precisely analyze the multidimensional information of proteins, thereby providing direct evidence for the translation activity of ncRNAs^23,24^. MS plays an irreplaceable role in biomolecular detection. Its core principle first involves digesting proteins into micropeptides using proteases, followed by ionizing these peptides and separating them according to their mass-to-charge ratios. This analytical process enables high-throughput identification of proteins and their post-translational modifications^25,26^. Compared with translational genomics, the identification of peptides by MS provides the most intuitive evidence that ncRNA is translationally active. When a specific peptide is detected during MS, it implies the presence of an endogenous ncRNA pair to code for it. This direct link from the peptide to the coding source confirms conclusively that ncRNA can participate in the translation process, which is a promising new direction for the subsequent study of the function and molecular mechanism of ncRNA^27^. In 2017, researchers identified a conserved and functional peptide encoded by lncRNA via MS that regulates mTORC1 activation and muscle regeneration in mammals^28^. It was also found that circBUB1B could encode a new protein circBUB1B_544aa, and the specific peptide segment at the circBUB1B_544aa cirRNA junction was detected by MS, which proved the authenticity of the circBUB1B encoding^29^.

However, the amount of ncRNA-encoding peptides was found to be very small in the process of MS identification, which was related to the loss during the preparation and purification of MS samples^30^. In addition, the low detection rate of ncRNA-encoded micropeptides is notably influenced by intrinsic biological factors. For example, some micropeptides are transiently expressed only at specific stages of skeletal muscle development, resulting in extremely low intracellular steady-state abundance^31^. Others, lacking stable spatial structures, are readily degraded by proteasomes and fail to accumulate to the threshold required for MS detection^32^. These inherent biological characteristics, combined with technical losses during sample processing, collectively contribute to the low detectability of ncRNA-encoded micropeptides in MS. It is important to note that the peptides encoded by ncRNAs are generally small in size, which makes MS detection challenging, resulting in error-prone results that do not fully satisfy the need for high sensitivity and high precision detection^33^. The unique circular structure of circRNAs also poses a challenge in the identification of circRNAs-encoded peptides. Because of its specificity, most circRNAs-encoded peptides have to be detected by focusing on specific peptide junctions. This requirement is extremely demanding in terms of equipment precision, environmental stability and reagent sensitivity, and any slight deviation may interfere with the accuracy of the results.

### Experimental validation

The translation process of ncRNAs is highly dependent on ribosomes, which play an irreplaceable role in the translation process and are directly related to the successful completion of ncRNAs translation. Sucrose density gradient centrifugation can be used to determine whether candidate ncRNAs bind to polyribosomes. The main steps of this method include preparing a continuous sucrose density gradient (for example, 10%-50%) and then loading the cell lysates onto the top of the gradient for centrifugation. Based on the difference in sedimentation coefficients between polyribosomes and free ribosomes/RNA, the RNA bound to polyribosomes can be distinguished from free RNA. In addition, translation inhibitors such as EDTA and puromycin can be used to analyze changes in ribosome binding to candidate ncRNAs before and after treatment^34,35^. Furthermore, beyond analyzing ribosome-RNA binding, this technique can separate ribosome polymers, facilitating studies on the coding potential of candidate ncRNAs^34–37^. The IRES is one of the important mechanisms driving the translation of ncRNAs. In some researches, the activity of IRES in ORFs is often detected with the help of dual-luciferase reporter vectors^27^. The primary approach involves inserting the IRES sequence associated with the ORF upstream of the firefly luciferase gene, with Renilla luciferase as an internal control. If there is an obvious increase in firefly luciferase activity, it indicates that the IRES is active and the ORF may possess coding potential^36,38–40^.

**Fig. 1:**
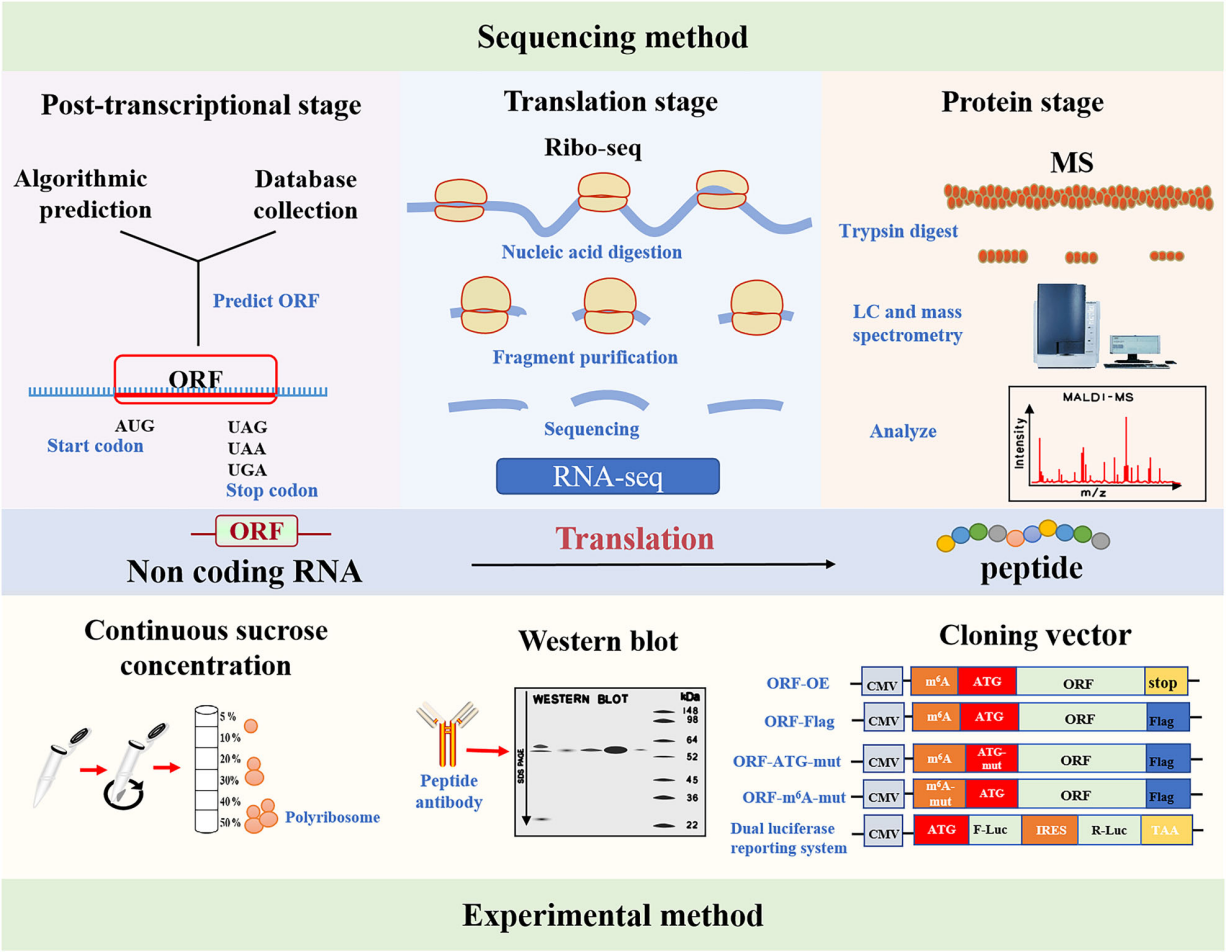
Methods for studying the coding activities of ncRNAs. Translatable ncRNAs can be identified through a combination of bioinformatic predictions and experimental validation. Bioinformatic approaches typically include ORF prediction, Ribo-seq, and MS analysis. Experimental techniques commonly involve ribosome gradient centrifugation, Western blot, and fusion-tag vectors.

Of course, other effective experimental methods include overexpressing or knocking down candidate ncRNAs, preparing specific antibodies against predicted peptides, and using western blot (WB) experiments to detect changes in peptide/protein expression^37^. In addition, m⁶A methylation is a key mechanism regulating ncRNA translation. This modification promotes efficient translation initiation of ncRNAs by recruiting translation initiation complexes and plays an important role in the ncRNA translation regulatory network. Moreover, constructing site-directed mutant plasmids of m⁶A modification sites and detecting the expression of encoded peptides/proteins can also verify the coding potential of ncRNAs^35^. To experimentally validate the coding potential of predicted ORFs, site-directed integration of epitope tags (for example, FLAG, HA) can also be achieved via CRISPR–Cas9. If the expression of the tagged protein is detected via assays including WB and immunofluorescence, and such expression is dependent on ORF translation, the coding activity of the target ORF is directly validated. Furthermore, this method enhances the efficiency of subsequent tracking, detection, and functional studies of the corresponding peptides or proteins^28,41^. Therefore, designing specific experiments for ncRNA ORF is also an effective means to prove the authenticity of ncRNA-encoded peptides/proteins (Fig. 1).

## Mechanisms that activate ncrnas translation

The translational activation of ncRNAs relies on three core components: sORFs, which constitute the basic coding units for peptides; IRES and m^6^A modifications. IRES and m^6^A modifications, located upstream of sORFs, play essential roles in regulating translation initiation. Together, these elements form a coordinated regulatory network governing the translation of ncRNA-encoded peptides.

### sORF

LncRNAs are a class of ncRNAs spanning 200 nucleotides (nt) in length, transcribed from intergenic regions. However, scientists identified 6170, 10,461, 30,521 and 23,599 sORFs in four legumes, namely bean, soybean, tribulus alfalfa and centella asiatica, by biological methods^42^. Since then, a large number of sORFs have been found in lncRNAs from zebrafish, mice and humans. Although sORFs are widespread, their actual detection rate remains low due to low micropeptide expression, poor stability and technical limitations. Moreover, most sORF-encoded micropeptides are expressed only under specific physiological conditions or in limited cell subtypes, hampering their detection by conventional bulk sequencing or MS. In 2013, Slavoff’s team used peptidomics to detect many of these peptides that are naturally occurring in living organisms^43^. After careful analysis, it was determined that some of these micropeptides are encoded by lncRNAs, and this finding was further validated by functional experiments^43^. In 2015, a study demonstrated that the myoregulin (MLN) peptide encoded by sORF in lncRNA in mice regulates calcium transport in the sarcoplasmic reticulum, thereby regulating muscle motility. However, when the sORF of this lncRNA was mutated, the MLN peptide was not detected^41^. In addition, several lncRNAs have been also identified in humans that encode proteins, such as the Humanin peptide that inhibits the proapoptotic protein BAX and thus protects cells from β-amyloid-mediated apoptosis^44^.

The maturation of miRNAs is produced by primary transcripts that are processed by a series of shearing processes by a variety of nucleases. miRNAs most primary transcripts are called pri-miRNAs, which vary in length from 300 to 1000 bases^45^. The vast majority of pri-miRNAs are transcribed by RNA polymerase II (Pol II). Only a few specific pri-miRNAs are transcribed by Pol III, which depends on their specific promoter elements. Pri-miRNA transcription driven by RNA Pol II has typical structural features: a 5′-cap structure at the 5′ end, a poly(A) tail at the 3′ end and at least one hairpin structure. These features facilitate the processing and translocation of pri-miRNAs, as well as their subsequent execution of gene regulatory functions in cells. Based on these characteristics, some researchers hypothesize that sORFs may be involved in encoding micropeptides within pri-miRNAs. Studies have shown that pri-miRNA171b from *Tribulus terrestris*-like alfalfa and pri-miR165a from Downy pine grass encode micropeptides of 9 amino acids (aa) and 18 aa respectively. miPEP172c was found to encode micropeptides in soybean by Jean-Malo Couzigou et al.^46^. In addition, pri-miR858a encodes a micropeptide that regulates flavonoid biosynthesis and arabidopsis development^47^. In grapevine plants, vvi-miPEP171d1 is expressed and plays a key role in the regulation of root development, profoundly affecting root growth and morphogenesis^48^. Currently, studies have shown that cytokinesis and passive diffusion are the main modes of entry of miPEP into cells, but the mechanism of translational translocation remains poorly understood. Researchers have also identified peptides encoded by pri-miRNA in the animals. Fang et al. found that miR-200a and miR-200b each encode a micropeptide in human cells, miPEP-200a and miPEP-200b, respectively, which provides a new perspective on the molecular regulatory mechanisms in human cells^49^. *Drosophila*-expressed miR-8 was also reported to express micropeptides that regulate development^50^.

**Fig. 2:**
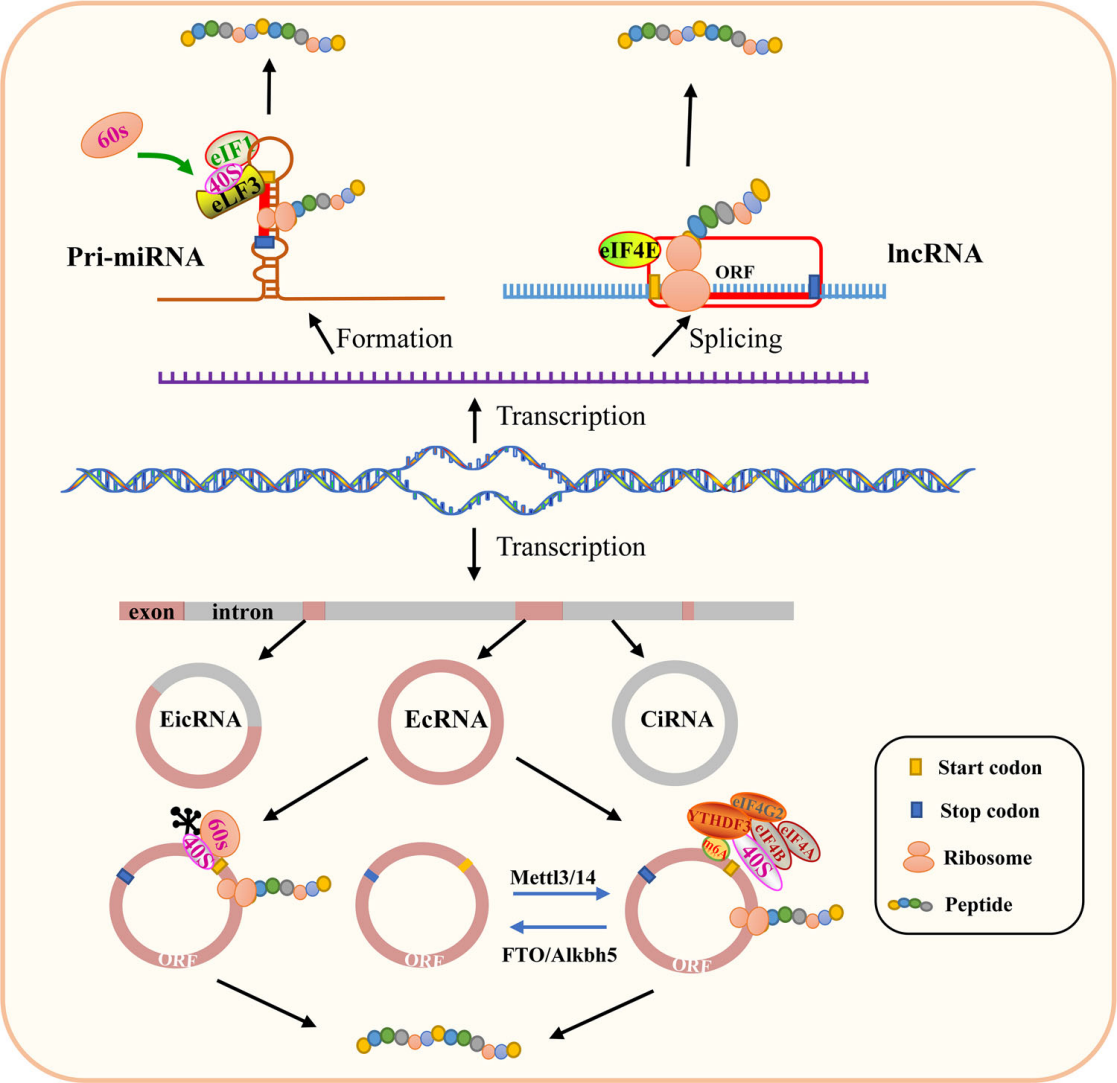
Translation driving mechanism of ncRNAs. The coding potential of translatable ncRNAs is primarily mediated through three major mechanisms: ORF, m6A methylation, and IRES.

These reports show that ncRNAs translation is widespread in both plants and animals and that the ORFs encoded are short, producing micropeptides mostly of less than 50 amino acids (Fig. 2). These ncRNA-encoded peptides/proteins are not ‘silent players’ but have unique and important biological functions such as cell signaling and regulation of gene expression.

### IRES

While sORF provides the basic coding unit, the IRES serves as the core element recognized by ribosomes to initiate translation. A classical noncapsid translation mechanism exists in DNA viruses and RNA viruses^51^, whereby translation is initiated by the recruitment of ribosomes to form translation complexes via IRES on the mRNA structure^52^. When eukaryotes receive stress and pressure, abnormalities in canonical translation are compensated for by noncapsid-dependent translation^53^. Consistent with the above translation mode, circRNA (as a circRNA without a cap structure) mainly relies on the IRES translation mechanism to initiate translation. In 1995, researchers found that circRNAs synthesized in vitro with IRES were able to be translated normally, whereas circRNAs without IRES structures were unable to express proteins. This strongly demonstrated the critical role of IRES for circRNA translation^54^. Subsequent researchers have also demonstrated by ribosome blotting that circRNAs can translate proteins efficiently via IRES in *Drosophila*^55^. In 2017, researchers identified a conserved circRNA, circ-ZNF609, in mouse and human frontal muscle cells. Further studies revealed that the untranslated region (UTR) of circ-ZNF609 enhances IRES-dependent translation. This phenomenon suggests that the incorporation of small fragments of endogenous intronic sequences into the test structure contributes to the enhancement of IRES activity^56^. These findings further support the mechanism by which IRES drives circRNA translation through recruiting ribosomes and provide a strong basis for an in-depth understanding of the translational regulation of circRNAs. Similarly, the translation of lncRNA can also be controlled through IRES. Researchers have found that the expression of lncRNA CTBP1-DT, is notably upregulated when cells experience DNA damage. After further investigation, it was found that CTBP1-DT could encode the DDUP protein within its 5′UTR through IRES during the DNA damage repair process, thus promoting the DNA damage repair process^57^. Taken together, these studies indicate that some ncRNAs for coding activities are IRES-dependent.

### m^6^A modifications

m^6^A modification is the most common post-transcriptional RNA modification of mRNA. It plays a crucial role in the maturation processing, degradation and maintenance of the stability of mRNA after its transcription^58^. However, in recent years, a large number of studies have pointed out that m^6^A modification is not exclusive to mRNAs, but also exists on ncRNAs^59^. Moreover, m^6^A modification also plays an important role in regulating the translation process of ncRNAs, which greatly expands people’s knowledge of the regulatory mechanism of RNA modification^60^. In 2017, scientists exploring the mechanism of circRNA translation used different known IRES with green fluorescent protein tags to synthesize circRNAs in vitro. They found an interesting phenomenon where some of the negative control circRNAs also emitted green fluorescence. Subsequent enrichment of the m^6^A methylation modification of RRACH (R = G/A, H = A, C, U) was found in the enrichment of the start codon of the negative control circRNAs^61^. By analyzing methylation RNA immunoprecipitation sequencing data, the researchers identified a large number of endogenous circRNAs with sites containing m^6^A modifications^62,63^. It was revealed that circRNAs can mediate translation of circRNAs through m^6^A methylation in addition to IRES to drive translation. Enhancement of the m^6^A demethyltransferase FTO inhibited circRNA translation; however, enhanced expression of the m^6^A methyltransferase METTL3/14 promoted circRNA translation^61^. The m^6^A methylated reading protein YTHDF3 can interact with the translation initiation factor eIF4G2 to recruit 40 s subunit ribosomes to form translation-associated complexes to promote circRNA translation^64,65^. It has also been shown that m^6^A methylation-mediated circRNA translation can be promoted under heat stress^66^.

It has been found that in addition to circRNAs, lncRNAs are also capable of initiating the translation process by means of m^6^A modification. For example, in patients with non-small cell lung cancer malignancies, the lncRNA AFAP1-AS1 encodes a conserved peptide of 90 amino acids, which is localized in the mitochondria. Further studies showed that this translational process is driven by the m^6^A modification on lncRNA AFAP1^67^. These results all indicate that m^6^A modification, as an important post-transcriptional regulatory modification, is one of the important ways of ncRNAs coding initiation. And these also demonstrate that circRNAs and lncRNA can mediate translation through m^6^A methylation on their structures.

## Regulation of skeletal muscle development by ncrnas encoding peptides

### lncRNA

Studies have shown that lncRNAs as ncRNA to play an important role in the regulation of skeletal muscle development^68–70^. However, as more and more evidence confirms the ability of ncRNAs to encode peptides/proteins, researchers have discovered that some lncRNAs can play a key role in skeletal muscle development by encoding peptides/proteins. During skeletal muscle contraction, the intracellular concentration of Ca^2+^ in skeletal muscle cells maintains a tightly regulated mechanism. The sarcoplasmic endoplasmic reticulum Ca^2+^ ATPase (SERCA) plays a key regulatory role in the maintenance of Ca^2+^ homeostasis^71^. However, SERCA activity is inhibited by myelin (SLN) and phosphoprotein (PLN), and their interactions have important implications for normal physiologic function of skeletal muscle^72^. Study in skeletal muscle have revealed that MLN, a micropeptide encoded by LINC00948, has structural similarities to SLN and PLN. MLN can interact with SERCA, ultimately exerting an inhibitory effect on the activity of the SERCA pump^41^. This finding contributes to the understanding of calcium homeostasis regulation and related physiological processes in skeletal muscle. Further knockdown of MLN expression in mice revealed a substantially higher efficiency of Ca^2+^ transfer in skeletal muscle cells and increased locomotor activity^41^. MLN is conserved in mammals, and the amino acid sequence homology between MLN in humans and mice exceeds 80%. Both regulate calcium homeostasis by binding to SERCA, indicating a high degree of functional conservation during evolution^41^. The researchers further also found that DWORF, a novel micropeptide consisting of 34 aa encoded by the lncRNA LOC100507537, acts as an activator of the SERCA pump in mouse skeletal muscle. In cardiomyocytes, the micropeptide DWORF reduces myocardial contraction time by activating SERCA pump activity. By contrast, in skeletal muscle, loss of DWORF leads to delayed Ca^2+^ clearance and reduced SERCA activity^73^. The mechanism by which DWORF acts is twofold: first, it competitively binds to phosphorylated protein, which is an inhibitor of the SERCA pump, and second, it reduces the degree of inhibition of the SERCA pump by phosphorylated protein, which in turn promotes the activity of the SERCA pump^73^. Researchers also found that the amino acid sequences of human DWORF and mouse DWORF are exactly the same. Experiments have all demonstrated that DWORF can enhance SERCA2a activity in both mouse in vivo models and human cardiomyocyte models^74^. Besides in mouse skeletal muscle, lncRNA-sarcolamban has been found to have coding potential in *Drosophila*, and its encoded proteins have high homology with SLN and PLN, but whether it really has the ability to encode and its functional mechanism are still unknown^75^.

In 2017, scientists identified a highly expressed and conserved polypeptide SPAR in human and mouse skeletal muscle, which is a novel 90 amino acid polypeptide encoded by a lncRNA LINC00961. Further studies revealed that SPAR localized in late lysosomes inhibits mTORC1 activity and recruitment of mTORC1 to the lysosomal membrane, ultimately promoting muscle regeneration in injured skeletal muscle^28^. In addition to studies in humans and mice, the researchers found that the lncRNA Six1 ORF2 encodes a peptide that was detected in chicken skeletal muscle^76^. The peptide binds to the Six1 promoter and activates the Six1 gene, which in turn promotes cell proliferation and division and ultimately regulates the development of chicken skeletal muscle^76^. These results further reveal that peptides or proteins encoded by lncRNAs may play crucial roles in the development of skeletal muscle in different species and provide new ideas and directions for cross-species studies on the mechanisms of skeletal muscle development.

### circRNA

circRNAs are circularly structured RNAs formed by variable shear reverse splicing of mRNA precursors and therefore do not have the traditional 3′UTR and 5′UTR regions^77^. It therefore also lacks the essential elements of eukaryotic translation. However, in 1995, scientists discovered that synthetic circRNAs could recruit translation-associated complexes in vitro and could actually initiate translation^54^. In 2014, researchers discovered that a natural viral-like circRNA could produce a 16 kDa peptide in an in vitro translation system^78^. Subsequent researchers have progressively identified translation-related structures in circRNAs in vivo, such as translation initiation codons and ORFs, and this evidence is gradually pointing to the coding potential of circRNAs in vivo^79,80^.

It has been reported that circRNAs that regulate muscle development can also encode polypeptides^81^. The ORF of circ-ZNF609 has the same start codon as mRNA, which regulates muscle cell proliferation and encodes polypeptides. In Duchenne muscular dystrophy muscles, the expression of circ-ZNF609 was distinctly down-regulated, and skeletal muscle cell proliferation was markedly reduced by 80% after interfering with circ-ZNF609, accompanied by down-regulation of the proliferation-related genes CDK1 and cyclinA2^56^. Studies on chicken skeletal muscle show that circEDC3 has a unique regulatory role. It inhibits the expression of genes related to the proliferation and differentiation of skeletal muscle cells, impedes the advancement of the cell cycle and the formation of myotube^82^. The internal sequence analysis of circEDC3 revealed that it is conserved among different species such as chicken, human, mouse, rat and pig, possessing different ORFs, IRESs, and m^6^A modification, which implies that circEDC3 is equipped to encode proteins^82^. The prerequisite is that it may have coding ability, but researchers have not further demonstrated the authenticity of its coding.

In 2023, researchers identified circTmeff1, a circRNA that exhibits high expression in multiple models of muscular dystrophy. In vitro and in vivo experiments proved that low expression of circTmeff1 effectively saved muscle atrophy. Further study found that circTmeff1 encoded a novel protein TMEFF1-339aa via IRES to promote muscle atrophy in mice in vitro^83^. Li et al. also identified a circRNA with the ability to encode circNEB by Ribo-seq in the longest dorsal muscle of the cattle. circNEB can encode a protein localized in the nucleus and cytoplasm at 907 aa (ref. ^84^). circNEB-encoded proteins were found to interact with SKP1 and TPM1 to promote the proliferation and differentiation of bovine myoblasts. In vivo, circNEB-encoded proteins induced regeneration of cardiotoxin-damaged muscles and promoted recovery of senescent atrophic muscles^84^. A new circRNA, circKANSL1L, was also identified in skeletal muscle of pigs. circKANSL1L encodes a 551 aa protein that interacts with Akt and ultimately activates the Akt–FoxO3 signaling pathway to promote C2C12 differentiation. However, neither of these studies in cattle and pigs has well revealed the mechanism of circRNA encoding with coding capabilities.

As the mechanisms of skeletal muscle development continue to be explored, a large number of studies have revealed that peptides encoded by newly discovered ncRNAs are able to regulate the expression of key muscle genes (Table 2). This discovery strongly suggests that these ‘alternative’ ncRNAs, which had been overlooked, play an essential role in the growth and development of skeletal muscle.

**Table 2:** NcRNAs encoding peptides in skeletal muscle system.

ncRNAs	Species	Translation initiation driver	Protein/peptide	Identification, methods and tools	Main functions
LINC00948	Human and mouse	sORF	MLN	Conserved analysis of ORF; in vitro transcription; FLAG tag fusion vector	MLN acts similar to PLNs and sarcolipins, acting directly on sarcoplasmic reticulum Ca^2+^-ATPase, preventing Ca^2+^ from entering the sarcoplasmic reticulum
LOC100507537	Mouse	sORF	DWORF	PhyloCSF; polyclonal rabbit antibody was prepared; EGFP tag fusion vector	DWORF peptide can neutralize SERCA inhibitors and reduce muscle contraction time
LINC00961	Human and mouse	sORF	SPAR	Tandem MS	SPAR can reduce mTORC1 activity and promote muscle regeneration
lncRNA MyolncR4	Mouse and zebrafish	sORF	LEMP	Construction of HA tag vector	LEMP promotes muscle formation and regeneration in mouse
circ-ZNF609	Human and mouse	IRES	/	Vector p-circ with a 3×FLAG tag; dual-luciferase reporting system; sucrose density gradient centrifugation	Controls myoblast proliferation
lncRNA-Six1	Chicken	sORF	lncRNA-Six1-ORF2	Vector pSDS-20218 with a 3×FLAG tag	Promots myoblast proliferation and migration
circEDC3	Chicken	IRES and m^6^A motifs were predicted	/	ORF were found in circEDC3	Inhibits myoblast proliferation, differentiation and apoptosis
circTmeff1	Mouse	IRES	TMEFF1-339aa	TransCirc database (https://www.biosino.org/transcirc/); 3×FLAG tag fusion vector; dual-luciferase reporting system	Promote muscle atrophy
circNEB	Cattle	/	circNEB-peptide	Ribo-seq; P-EGFP-N1 fusion protein vector	circNEB-peptide promotes the proliferation and differentiation of bovine myoblasts through ubiquitination and myoblast fusion by directly interacting with SKP1 and TPM1
CircKANSL1L	Pig	/	KANSL1L-551aa	KANSL1L-551aa with 3×FLAG tag	The circKANSL1L protein could activate the Akt–FoxO signaling pathway to regulate C2C12 differentiation

## Potential clinical application of ncRNAs encoding peptides in muscular diseases

**Fig. 3:**
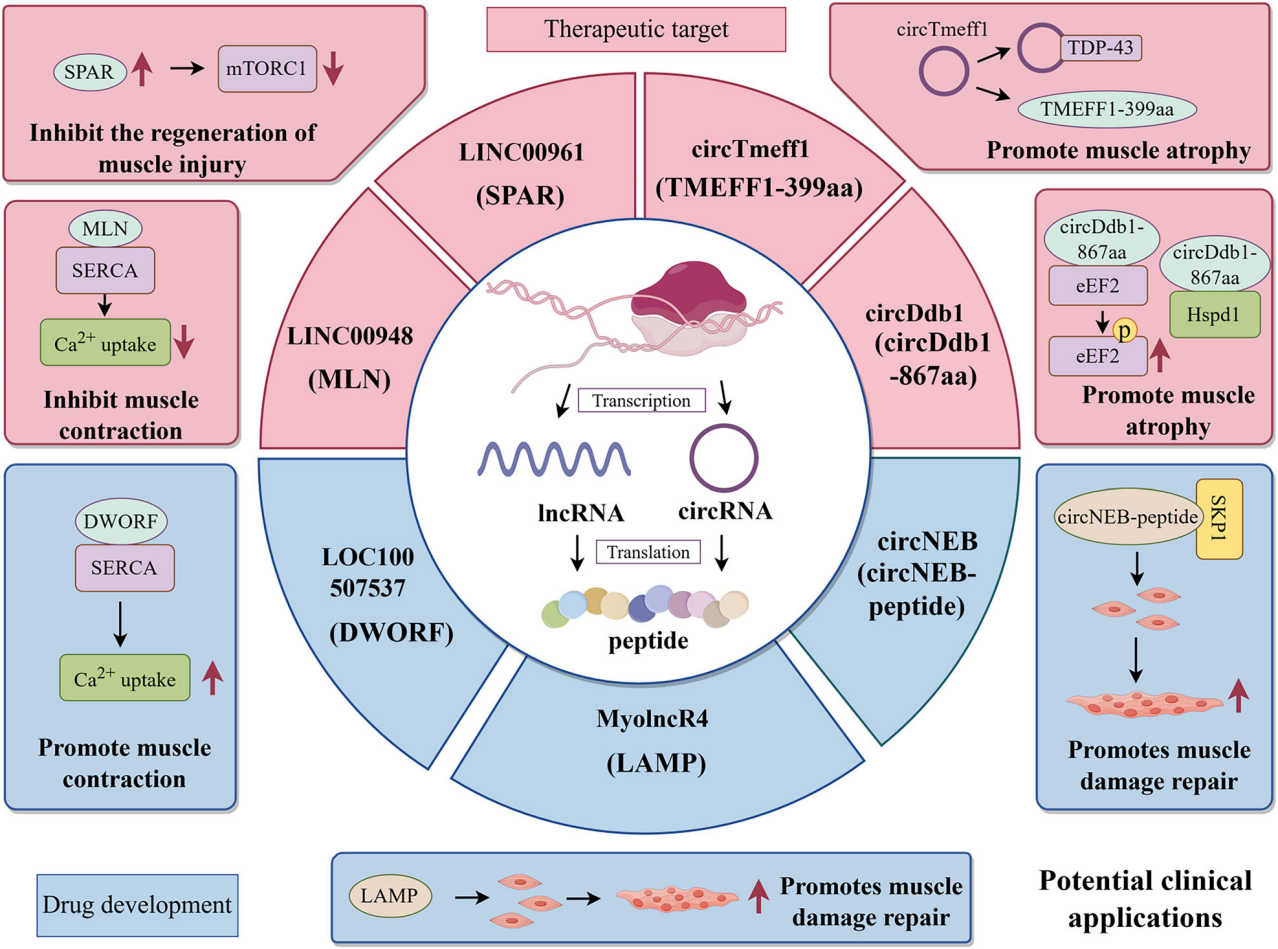
The potential application of ncRNA-encoded peptides. This figure was drawn by Figdraw. Peptides/proteins encoded by ncRNAs play important regulatory roles in muscle development, atrophy, and injury repair, holding promising potential for future clinical therapeutic applications.

Numerous research results have shown that ncRNAs and the peptides/proteins they encode play a key role in the regulation of physiological processes in the body. Currently, many research teams have gradually applied ncRNA-encoded peptides in cancer therapy, and the results of this exploration also provide new ideas and directions for applying ncRNA-encoded peptides to the development of clinical therapeutic programs for muscle diseases (Fig. 3).

### Therapeutic targets

It has been shown that circTmeff1 and circDbd1 encoded proteins have a promotional effect on muscle atrophy, and muscle atrophy can be effectively rescued in denervated mice by targeting to reduce circTmeff1 and circDbd1 expression, suggesting that the circTmeff1 and circDbd1 encoded proteins are potential targets for the treatment of muscle atrophy^83,85^. Failure of Ca^2+^ signaling in muscle is the cause of many muscle diseases. A study found that lncRNA-encoded MLN is a SERCA inhibitory micropeptide, and MLN knockdown in mice effectively reduced SERCA activity and enhanced skeletal muscle contraction in mice, suggesting that MLN has the potential for skeletal muscle performance-enhancing therapy^41^. In addition to this, deletion of the expression of SPAR, a novel peptide encoded by mouse lncRNA LINC00961, was given to efficiently promote muscle regeneration after injury in vivo. This series of findings suggests that peptides/proteins encoded by ncRNAs could be potential targets for the treatment of muscle atrophy and injury. In-depth investigation of ncRNA-encoded peptides as therapeutic agents for skeletal muscle diseases is of great value and may open up new pathways for the treatment of related diseases.

### Drug development

Currently, gene therapy and antibody therapy are the mainstays of muscle disease treatment. Peptides/proteins with low side-effects as drug therapy are also gradually being considered as a means of treatment. Overexpression of circNEB-peptide by injection of circNEB plasmid into cardiotoxin-injured mouse skeletal muscle promotes the repair of injured muscle, suggesting that circRNA can be used as an RNA therapy for the treatment of injured muscle by expression of peptide^84^. Some studies have also shown that the functional overexpression of DWORF in mice using gene editing technology effectively increases SERCA activity to enhance the contraction of cardiomyocytes, suggesting that DWORF can be used to treat heart disease by increasing cardiac contractility^73^. Wang et al. found that lncRNA MyolncR4 encodes the peptide LEMP in various vertebrates. LEMP is conditionally knocked out resulting in impaired muscle regeneration and reduced muscle strength, which strongly suggesting that LEMP has a crucial role in myogenesis as well as in the recovery of damaged muscles^31^. These researches reveal that ncRNAs-encoded peptides have the potential to be developed into therapeutic drugs that can be used to improve the contractile function of damaged muscles and the heart, providing a new direction for therapeutic options for muscle diseases, which is expected to bring benefits to many patients. Although the above studies have confirmed the potential of ncRNA-encoded peptides/proteins in repairing damaged muscle and improving cardiac contractile function, their clinical translation remains constrained by core bottlenecks, including low delivery efficiency, poor in vivo stability and insufficient tissue targeting. Currently, lipid nanoparticles and adeno-associated viruses can effectively protect nucleic acids from degradation, enhance tissue specificity and improve cellular uptake efficiency^86,87^. However, research on ensuring the translation efficiency of coding ncRNAs remains relatively limited^88^. Therefore, optimizing the design of ncRNA delivery vectors and ensuring their translation efficiency are critical for advancing the clinical translation of ncRNA-encoded peptide therapies.

## Conclusions and future perspectives

Numerous studies have shown that ncRNAs play a key role in skeletal muscle development. Previously, most studies have focused on the interactions between ncRNAs and proteins. However, in recent years, some researchers have begun to focus on the ORFs in ncRNAs, which are capable of encoding polypeptides and performing specific functions, opening up a new way of thinking for the study of ncRNAs. But currently, most of the studies on coding of ncRNAs are focused on cancer-related studies, and the relevant prediction databases and websites to study the coding activity of ncRNAs in different species are not yet complete. Second, since the ORFs of ncRNAs are mainly composed of exons, which are consistent with the mRNA sequences of the source genes, it increases the difficulty of peptide identification. In the identification of ncRNAs-encoded peptides, there are problems such as micropeptide size, low abundance and poor stability, which mask the existence of more ncRNAs-encoded peptides. Thus, the number of ncRNAs with coding potential and the associated functions of their coding peptides remain unknown.

In this Review, the main translation mechanism of ncRNAs encoding peptides, the research progress of encoding peptides involved in skeletal muscle development, and the potential clinical applications in muscle diseases are reviewed. This opens up a whole new path of exploration to improve the research system of skeletal muscle development and to explore potential solutions for the treatment of muscle diseases. However, they also give us some thoughts, such as whether ncRNAs identified earlier with stronger functions also have the ability to code and perform their main functions through coding peptides? What are the functions of ncRNAs with coding potential and their coding peptides during skeletal muscle development? How is the translation of ncRNAs dynamically regulated in the skeletal muscle system and what factors affect their translation? These are all areas that still need further exploration. In the future, can functional ncRNAs-encoded peptides be synthesized into proteins by synthetic means and transferred into the body via adeno-associated viruses or nanoparticles to perform their functions in the clinic? The functional verification of a large number of ncRNA-encoded peptides in skeletal muscle diseases has only been carried out in mice, and it still needs a lot of validation as a therapeutic agent for skeletal muscle diseases in the future. How to ensure the stability of ncRNA-encoded peptides during treatment still needs further research. In addition, the development of delivery systems for peptides encoded by ncRNAs is a challenging task. At the same time, circRNAs are more stable than mRNA vaccines and cause less immune stress. This has led to the question of whether endogenous and synthetic circRNAs with coding ability can be investigated as an alternative to mRNAs for use in vaccines.

More and more ncRNAs have been gradually verified to encode functional peptides, and their detection technologies, functional verification methods and future clinical applications are worthy of further investigation.
